# Temporal changes in breast cancer lifetime risk and implications for personalised surveillance in high-risk women

**DOI:** 10.1186/s13244-026-02355-9

**Published:** 2026-07-22

**Authors:** Machteld Keupers, Sam Nijssen, Willem Sarkol, Yao-Kuan Wang, Lesley Cockmartin, Hans Wildiers, Hilde Bosmans, Chantal Van Ongeval

**Affiliations:** 1 Department of Radiology, University Hospital Leuven, Leuven, Belgium; 2 University Hospital Leuven, Multidisciplinary Breast Centre, Leuven, Belgium; 3 Department of Imaging and Pathology, KU Leuven, Leuven, Belgium; 4 Department of Medical Physics, University Hospital Leuven, Leuven, Belgium

**Keywords:** Breast neoplasms, Risk assessment, Risk factors

## Abstract

**Objectives:**

To evaluate temporal changes in breast cancer lifetime risk (BC-LTR) over a 10-year period among women with clinically elevated risk but without known pathogenic genetic mutations, using the International Breast Cancer Intervention Study (IBIS) breast cancer risk evaluation tool, and to assess implications for personalised surveillance strategies.

**Materials and methods:**

In this retrospective study, women referred for elevated risk assessment in 2014 underwent BC-LTR estimation using IBIS (v8.0b). Risk was recalculated in 2024 using the same model without recalibration. Women who developed breast cancer, underwent bilateral mastectomy, died during follow-up, were older than 85 years in 2024, or carried pathogenic genetic mutations were excluded, as IBIS BC-LTR recalculation is not applicable in these contexts. Risk factor temporal changes were evaluated using paired statistics. A linear mixed model assessed predictors of IBIS BC-LTR and their association with temporal changes.

**Results:**

Among 362 eligible women, mean IBIS BC-LTR decreased from 23% in 2014 to 19% in 2024 (mean absolute change = 6.5, *p* < 0.001). Risk group reclassification occurred in 44%: 36% shifted to a lower risk category and 8% to a higher category. Increase in IBIS BC-LTR was associated with increasing density (*p* < 0.0001), higher BMI (*p* = 0.002), current HRT use (*p* = 0.007), and additional affected relatives (*p* < 0.03), whereas downward reclassification was mainly linked to decreasing density (*p* < 0.0001).

**Conclusion:**

IBIS BC-LTR estimates change over time in women with elevated clinical risk. Periodic reassessment may better align estimated risk with tailored screening strategies, supporting more precise, individualised surveillance.

**Critical relevance statement:**

Variation in IBIS BC-LTR scores over time highlights the need for periodic recalculation to improve individual risk stratification and guide more appropriate, personalised imaging and screening pathways.

**Key Points:**

- Breast cancer risk models identify women most likely to benefit from supplemental screening.
- IBIS BC-LTR estimates changed over 10 years, reclassifying over 40% of women with elevated baseline risk into a different risk category. Periodic IBIS BC-LTR recalculation is essential to optimise risk stratification and screening pathways.

**Graphical abstract:**

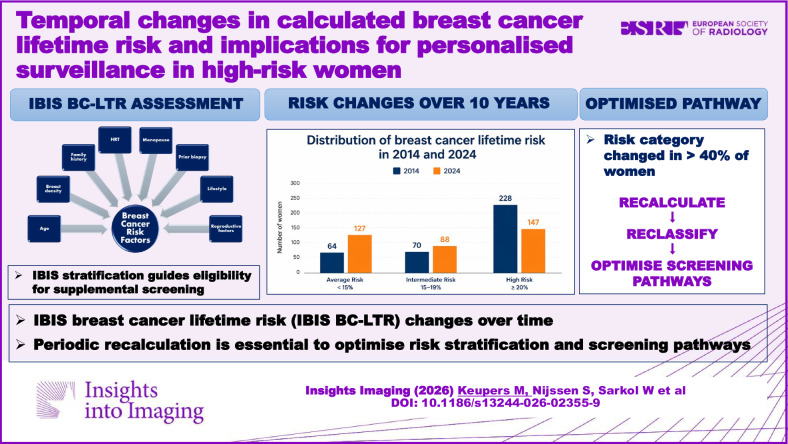

## Introduction

Breast cancer is the most common invasive malignancy among women worldwide and the leading cause of cancer-related mortality in Western countries [1]. Pathogenic germline variants in high-risk genes, such as *BRCA1* and *BRCA2*, account for only a small proportion of breast cancer cases. Non-hereditary risk factors include personal, lifestyle, and medical history-related risk factors [2, 3].

Early detection reduces breast cancer morbidity and mortality, which has led many countries to implement population-based mammographic screening programmes [1, 4, 5]. International hereditary breast and ovarian cancer (HBOC) screening guidelines exist for gene mutation carriers, including annual breast MRI [6, 7]. In line with European recommendations, women are commonly classified as high risk when the predicted BC-LTR is ≥ 20%, most often calculated using validated models such as the IBIS (Tyrer–Cuzick) tool.

Women at increased breast cancer risk (IBIS BC-LTR ≥ 20%) are preferentially recommended to undergo contrast-enhanced imaging, such as breast MRI, with ultrasound considered only when MRI is unavailable or contraindicated, as its additional cancer detection beyond mammography is more limited [3, 8]. For women at intermediate breast cancer risk (IBIS BC-LTR 15–19%), guidelines for supplemental screening are unclear. In Belgium, supplemental MRI is reimbursed only for women with an IBIS BC-LTR ≥ 30%. Additionally, capacity constraints limit the use of breast MRI for high-risk women with IBIS BC-LTR ≥ 20% [8–10].

Breast cancer risk prediction models combine population risk with individual risk factors to guide personalised surveillance [10–12]. At our institution, the IBIS (Tyrer-Cuzick) Breast Cancer Risk Evaluation model (v8.0b)—routinely used for over 15 years as one of the most validated tools for estimating BC-LTR—is applied to stratify who benefits most from supplemental imaging [13]. IBIS is suitable for women up to 84 years of age and integrates family history, breast density, hormonal and reproductive factors, and prior benign disease [10, 13–15].

This study builds upon prior research in using IBIS to objectively estimate the BC-LTR for women with a clinically elevated breast cancer risk due to positive family history and/or dense breasts [16]. IBIS risk calculator v8.0b was used to prove that this group of women, referred to our institution in 2014 for clinically elevated breast cancer screening outside population-based screening, had an elevated IBIS BC-LTR based on the electronic patient files (EPF) details. We concluded that this subgroup is heterogeneous, ranging from slightly higher-than-average IBIS BC-LTR to high IBIS BC-LTR. No clear guidelines for supplemental screening exist for this specific subgroup of asymptomatic women with clinically elevated breast cancer risk due to dense breasts, and/or positive family history, and/or other personal risk factors, but without known pathogenic genetic mutation.

The current study examines how IBIS BC-LTR scores have changed over the past decade in this subgroup of women with clinically elevated breast cancer risk due to dense breasts and/or positive family history but without known genetic mutation, and whether these changes have an influence on their proposed personalised breast cancer screening strategies.

## Material and methods

### Study design and participants

This institutional review board-approved retrospective study (S69370) included all women referred to the multidisciplinary breast centre of University Hospitals Leuven, Belgium, in 2014 for assessment of clinically elevated breast cancer risk. Referral was based on the presence of dense breast tissue (BI-RADS C/D) and/or a positive family history of breast and/or ovarian cancer, and/or other personal risk factors, in the absence of known pathogenic genetic mutations. The present analysis builds on this original cohort and evaluates changes in estimated IBIS BC-LTR over a 10-year period.

Mammographic breast density was assessed visually according to the ACR BI-RADS® Atlas (5th edition) via consensus reading of two breast radiologists [17].

Women were excluded from the present analysis from both datasets of 2014 and 2024 if they had a diagnosis of breast cancer prior to baseline assessment or developed breast cancer during the 10-year follow-up, underwent bilateral prophylactic mastectomy, died during the follow-up period, were older than 85 years in 2024, or were found to carry a pathogenic genetic mutation during follow-up. These exclusions were applied because the (re)calculation of IBIS BC-LTR is not valid in these situations.

All data required to recalculate the IBIS BC-LTR were retrospectively extracted from EPF at the University Hospitals Leuven and analysed for each woman individually. The analysis focused on IBIS BC-LTR scores, as 10-year IBIS scores were not available for 2014. The following variables were collected: current age, age at menarche and menopause [18], height, weight, mammographic breast density, hormone replacement therapy (HRT) use, parity, age at first childbirth, prior breast biopsy, personal history of ovarian cancer, family history of breast and ovarian cancer in first- and second-degree relatives (including age at onset and bilaterality), Ashkenazi ethnic background, and results of genetic testing for the individual and family members. Family history was defined as the presence of breast and/or ovarian cancer in one or more first- and/or second-degree relatives. Missing variables were not imputed. No default values or assumptions were applied to compensate for missing variables.

BC-LTR was calculated using the IBIS (Tyrer–Cuzick) model v8.0b at both time points. The same model version and assumptions were used in 2014 and 2024, without recalibration or modification. In clinical practice at our institution, recalculation of IBIS BC-LTR is typically considered when relevant risk factors change.

### Statistical analysis

Descriptive statistics were used to summarise participant characteristics from the 2014 and 2024 cohorts in Table 1. Paired comparisons between 2014 and 2024 cohorts were performed using paired *t*-tests for continuous variables and Stuart–Maxwell tests for categorical variables. Linear mixed model regression analyses were performed to identify variation in factors associated with a change in breast cancer risk and their upward or downward risk group reclassification, including changes in breast density, BMI, HRT use, and family history. Statistical significance was set at *p* < 0.05. All statistical analyses were performed using SPSS v30.0 (IBM).

**Table 1:** Overview of the participants’ characteristics required for calculation of the IBIS BC-LTR score in 2014 and 2024

2014				2024			
Characteristic	*n*	Missing data	Mean ± SD (range)	Characteristic	*n*	Missing data	Mean ± SD (range)
Age, mean ± SD (range)	362	0	49 ± 7.2 (37–74)	Age, mean ± SD (range)	362	0	59 ± 7.2 (47–84)
30–39	8 (2%)			30–39	0 (0%)		
40–49	208 (58%)			40–49	8 (2%)		
50–59	113 (31%)			50–59	208 (57%)		
60–69	27 (7%)			60–69	113 (31%)		
70–79	6 (2%)			70–79	27 (7%)		
> 80	0 (0%)			> 80	6 (2%)		
Age at menarche	174	188	13 ± 1.6 (9–17)	Age at menarche	174	188	13 ± 1.6 (9–17)
9	1 (1%)			9	1 (1%)		
10	6 (3%)			10	6 (3%)		
11	28 (16%)			11	28 (16%)		
12	32 (18%)			12	32 (18%)		
13	39 (22%)			13	39 (22%)		
14	46 (26%)			14	46 (26%)		
15	11 (6%)			15	11 (6%)		
16	8 (5%)			16	8 (5%)		
17	3 (2%)			17	3 (2%)		
BMI (kg/m^2^)	346	16	25.3 ± 4.7 (16–45.8)	BMI (kg/m^2^)	348	14	25.5 ± 4.7 (14.3–45.8)
Underweight (≤ 18.4)	5 (1%)			Underweight (≤ 18.4)	6 (2%)		
Normal weight (18.5–24.9)	184 (53%)			Normal weight (18.5–24.9)	175 (50%)		
Overweight (25–29.9)	100 (29%)			Overweight (25–29.9)	113 (32%)		
Obesity (≥ 30)	57 (16%)			Obesity (≥ 30)	54 (16%)		
Age at menopause	161	201	50.9 ± 4.4 (32–59)	Age at menopause	200	162	50.8 ± 4.3 (32–60)
Menopausal status	320	42		Menopausal status	312	50	
Pre	83 (26%)			Pre	48 (15%)		
Peri	32 (10%)			Peri	19 (6%)		
Post	205 (64%)			Post	245 (79%)		
Breast density	362	0		Breast density	362	0	
BI-RADS A (extremely fatty)	20 (5%)			BI-RADS A (extremely fatty)	33 (9%)		
BI-RADS B (scattered density)	119 (33%)			BI-RADS B (scattered density)	147 (41%)		
BI-RADS C (heterogeneous density)	133 (37%)			BI-RADS C (heterogeneous density)	163 (45%)		
BI-RADS D (extremely dense)	90 (25%)			BI-RADS D (extremely dense)	19 (5%)		
Parity	357	5		Parity	357	5	
Nulliparous	58 (16%)			Nulliparous	58 (16%)		
Parous	299 (84%)			Parous	299 (84%)		
Age at first childbirth	223	139	28.4 ± 4.4 (17–41)	Age at first childbirth	223	139	28.4 ± 4.4 (17–41)
< 25	37 (17%)			< 25	37 (17%)		
25–29	106 (47%)			25–29	106 (47%)		
≥ 30	80 (36%)			≥ 30	80 (36%)		
HRT	351	11		HRT	351	11	
Current user	11 (3%)			Current user	39 (11%)		
Never	319 (91%)			Never	274 (78%)		
Stopped < 5 y ago	9 (3%)			Stopped < 5 y ago	12 (4%)		
Stopped > 5 y ago	12 (3%)			Stopped > 5 y ago	26 (7%)		
Prior biopsy	362	0		Prior biopsy	362	0	
None	342 (94%)			None	295 (81%)		
Yes, but not proliferative (B2)	18 (5%)			Yes, but not proliferative (B2)	64 (18%)		
Hyperplasia (no atypia)	0 (0%)			Hyperplasia (no atypia)	1 (0%)		
B3 (other)	2 (1%)			B3 (other)	1 (0%)		
Atypical hyperplasia	0 (0%)			Atypical hyperplasia	1 (0%)		
Genetic testing	43	319		Genetic testing	43	319	
Tested normal	23			Tested normal	43		
First-degree relative with breast/ovarian cancer	362	0	Average number	First-degree relative with breast/ovarian cancer	362	0	Average number
Yes	311 (86%)		1.24	Yes	321 (89%)		1.31
No	51 (14%)			No	41 (11%)		
Second-degree relative with breast/ovarian cancer	362	0	Average number	Second-degree relative with breast/ovarian cancer	362	0	Average number
Yes	226 (62%)		1.88	Yes	242 (67%)		2.09
No	136 (38%)			No	120 (33%)		

## Results

The study population comprised 362 women with elevated IBIS BC-LTR based on breast density (BI-RADS C/D), and/or family history, and/or other personal risk factors, but without a known genetic mutation. IBIS BC-LTR was calculated for 362 women in 2014 and 2024, with missing variables excluded from the model. Table 1 presents all parameters used to calculate IBIS BC-LTR in 2014 and 2024, together with the extent of missing data. No default values or assumptions were applied to compensate for missing variables. In a fraction of women, the data on age at menarche (*n* = 188), body mass index (BMI) (*n* = 16), age at menopause (*n* = 46), parity (*n* = 5), breast density (*n* = 1), or HRT (*n* = 11) were not available in the EPF. For 50 women, the menopausal status was unknown, which may be due to the information not being recorded in the EPF or because the status was unclear due to the use of contraception such as the pill or an intrauterine device.

From the initial cohort of 2014, a total of 32 women with breast cancer diagnosis prior to baseline or during follow-up were excluded (23 invasive ductal carcinoma, 1 invasive lobular carcinoma, 8 ductal carcinoma in situ) together with 33 identified pathogenic variants (out of the 89 eligible for HBOC testing) [19] (BRCA1 (*n* = 7), BRCA2 (*n* = 15), CHEK 2 (*n* = 6), STK11 (*n* = 1), GCH1 (*n* = 1), MSH6 (*n* = 1), JAK2 (*n* = 1), and PALB2 (*n* = 1)). IBIS v8.0b is not intended to calculate BC-LTR in the presence of breast cancer or known high-risk mutations. For women with confirmed pathogenic variants, other risk calculation models such as CanRisk (implementing the BOADICEA model) are methodologically more appropriate.

Mean ± standard deviation (std) age was 49 ± 7.2years (range 37–74) in 2014 and 59 ± 7.2years (range 47–84) in 2024; 68% were postmenopausal at follow-up in 2024.

**Fig. 1:**
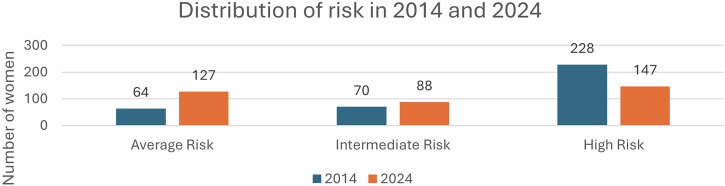
Distribution of IBIS BC-LTR in 2014 and 2024 (average risk < 15%, intermediate risk 15–19%, high risk ≥ 20%)

Mean IBIS BC-LTR decreased from 23% in 2014 to 19% in 2024 (mean absolute change = 6.5, *p* < 0.001). Overall, 56% of women remained in the same risk category, 8% were reclassified upward, and 36% downward (Fig. 1).

Over 10 years, BMI remained stable (*p* = 0.318), whereas breast density categories and IBIS risk categories changed significantly (*p* < 0.001 and *p* = 0.009, respectively).

**Fig. 2:**
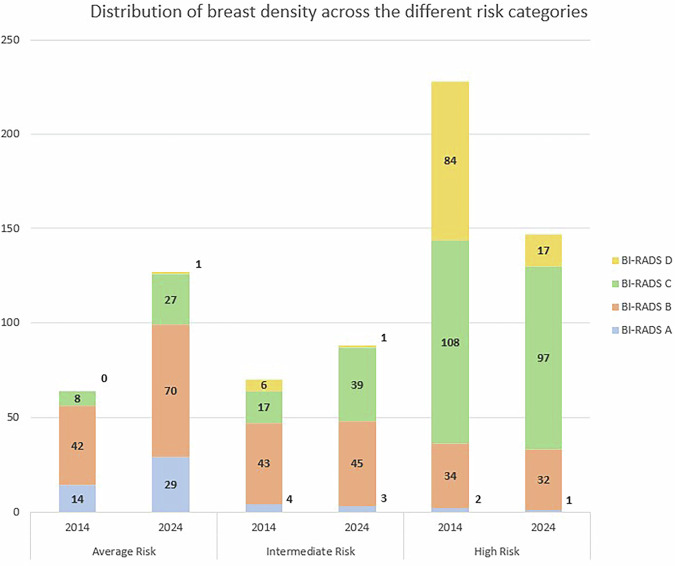
Changes in breast density (BI-RADS) category between 2014 and 2024

Breast density is known to be a major risk factor for breast cancer, and density changes influence the IBIS score. Over the 10-year study period, 202 women (56%) remained in the same BI-RADS density category, 138 (38%) showed a decrease in density, and 22 (6%) showed an increase. In 2024, density distribution was BI-RADS A 9%, B 41%, C 45% and D 5%, with a significant redistribution compared to 2014 (*p* < 0.001), with 6%, 33%, 37% and 25%, respectively. Characterised by a marked decline in BI-RADS D and an increase in BI-RADS B/C (Fig. 2). Most women were parous (84%; mean age at first birth 28 years). A family history of breast and/or ovarian cancer was present in 85.9% (first-degree) and 62.4% (second-degree relatives) at baseline.

Changes in IBIS risk categories between 2014 and 2024 are summarised in Table 2. Of the 362 women included, 202 (56%; 202/362) remained in the same risk group. Twenty-nine women (8%; 29/362) were reclassified into a higher risk category, including 12 (41%; 12/29) who moved from average to intermediate risk and 17 (59%; 17/29) from intermediate to high risk. In contrast, 131 women (36%; 131/362) were reclassified into a lower risk category: 56 (43%; 56/131) from high to intermediate risk, 42 (32%; 42/131) from high to average risk, and 33 (25%; 33/131) from intermediate to average risk with an overall significant redistribution (*p* < 0.001).

**Table 2:** Changes in IBIS BC-LTR score-based risk groups between 2014 and 2024

Same risk group in 2024	*n* = 202 (56%)
Higher risk group in 2024	*n* = 29 (8%)
From the average to the intermediate risk group From the average to the high-risk group From the intermediate to the high-risk group	*n* = 12 (41%) *n* = 0 *n* = 17 (59%)
Lower risk group in 2024	*n* = 131 (36%)
From the high to the intermediate risk group From high to average risk group From the intermediate to the average risk group	*n* = 56 (43%) *n* = 42 (32%) *n* = 33 (25%)

A linear mixed model evaluating effects of BMI, breast density, HRT usage, menopausal status and first/second degree family history on the IBIS BC-LTR for the baseline data showed that breast density was the strongest influencing input parameter of IBIS BC-LTR; BI-RADS A was associated with an approximately 23 percentage-point lower risk compared with BI-RADS D, for BI-RADS B and C, a decrease of 15 and 8 percentage points, respectively. was found (*p* < 0.0001). BMI significantly influenced IBIS BC-LTR scores, with each 1 kg/m² increase in BMI associated with a 0.5 percentage point increase in IBIS BC-LTR (*p* < 0.0001). Each additional first-degree relative diagnosed with breast cancer after 2014 was associated with a 5.4 percentage point increase in IBIS BC-LTR (*p* = 0.01), compared to a 1.4 percentage point increase per additional second-degree relative (*p* = 0.03).

As expected, an increase in BMI (per 1 kg/m²) was associated with a decrease in breast density category, with the odds being 16% lower in 2014 (OR = 0.84) and 17% lower in 2024 (OR = 0.83) (*p* < 0.0001). Another observed correlation was between the usage of HRT and breast density, where current HRT users had higher odds of being in a higher breast density category compared to those who stopped HRT for more than 5 years (OR = 4.4, *p* = 0.003).

Longitudinal changes in IBIS BC-LTR were primarily driven by changes in breast density (*p* < 0.0001). Increase in IBIS BC-LTR was associated with increasing density (*p* < 0.0001), higher BMI (*p* = 0.002), current HRT use (*p* = 0.007), and additional affected relatives (*p* < 0.03), whereas downward reclassification was mainly linked to decreasing density (*p* < 0.0001).

The above regression analysis confirms the sensitivity of the IBIS model to changes in its input variables.

## Discussion

This study evaluated whether IBIS BC-LTR scores changed over a 10-year period in women with elevated baseline risk due to positive family history and/or dense breasts, but without know genetic mutation. This subgroup is heterogeneous (varying from slightly-higher-than-average IBIS BC-LTR to high IBIS BC-LTR), and clear guidelines for supplemental screening are lacking.

Women with IBIS BC-LTR ≥ 20% are preferentially recommended to undergo supplemental contrast-enhanced imaging, particularly breast MRI, with ultrasound reserved for cases in which MRI is unavailable or contraindicated [8]. Although MRI has superior sensitivity compared with ultrasound, its implementation in supplemental screening in high-risk populations is constrained by resource availability and different reimbursement policies internationally.

The definition of intermediate or elevated BC-LTR is not standardised across studies and healthcare systems. Differences exist not only in the risk models and risk thresholds used, but also in the populations studied.

In Belgium, reimbursement for supplemental MRI is restricted to women with IBIS BC-LTR ≥ 30%, and limited MRI availability further constrains access for women with BC-LTR ≥ 20% [8, 9]. Consequently, the screening strategy evaluated in this study may differ from approaches adopted in healthcare systems with broader access to screening MRI.

Among 362 women, 202 (56%) remained in the same risk group, 29 (8%) moved to a higher category, and 131 (36%) moved to a lower one. Upward reclassification was primarily driven by changes in IBIS model input variables, particularly increases in breast density, the number of affected relatives, HRT use, and BMI, whereas downward reclassification was largely attributable to decreases in breast density. These changes directly affected supplemental screening recommendations [7–9]: women moving from average to intermediate risk (IBIS BC-LTR 15–19%) (*n* = 12) may benefit from supplemental imaging, and those shifting from intermediate to high risk (IBIS BC-LTR ≥ 20%) (*n* = 17) may become eligible for MRI. After 10 years, 98 of 362 women with clinically elevated risk (defined by dense breasts and/or a positive family history but without a known genetic mutation) shifted from a high-risk group to an intermediate (*n* = 56) or average (*n* = 42) risk group, making MRI screening no longer indicated according to our national policy.

Breast density is a major component of the IBIS model and an established risk factor for breast cancer. Because breast density generally decreases with age and increasing BMI, but may increase with HRT use, changes in breast density can substantially influence recalculated IBIS risk estimates over time. Ohmaru et al reported that approximately 25% of women experience reduced breast density over 10 years [20]. In our clinically elevated breast cancer risk cohort, 38% showed a decrease in density class, primarily among women with initially dense breasts (BI-RADS C/D). Because dense tissue increases breast cancer risk and decreases mammographic sensitivity, changes in density can influence risk assessment and early detection [1–3].

Other changes in IBIS BC-LTR estimates over time were partly attributable to changes in risk factor parameters, such as HRT use, BMI, and family history. Current HRT use, particularly combined oestrogen-progestogen therapy, is associated with an increased risk of breast cancer, whereas past use carries less risk [13]. Garcia-Estevez et al reported that a higher BMI, especially after menopause, is linked to an increased risk of invasive breast cancer [21]. Among 211 women with complete data, mean BMI remained stable (25.3 kg/m^2^ in 2014 vs 25.5 kg/m^2^ in 2024) in this cohort.

As our study population was selected based on a positive family history of breast cancer but without know genetic mutation, participants already represented a group at increased baseline risk. Nevertheless, the degree of familial burden remained an important driver of IBIS BC-LTR estimates. Each additional first-degree relative diagnosed with breast cancer after 2014 was associated with a 5.4 percentage point increase in IBIS BC-LTR (*p* = 0.01), compared to a 1.4 percentage point increase per additional second-degree relative (*p* = 0.03).

Reliance on a single baseline risk estimate may result in misalignment between estimated risk and recommended surveillance strategies. Consequently, periodic recalculation of IBIS BC-LTR, following relevant changes in risk factors, may improve alignment between estimated risk and screening recommendations. It can help maintain accurate, personalised screening strategies, ensuring appropriate use of MRI while avoiding unnecessary imaging in women whose risk decreases. Updated assessments require accurate data and clinical resources. Structured patient questionnaires capturing all IBIS parameters are necessary to improve reliability.

Risk reassessment and reclassification may have psychological implications for women undergoing long-term surveillance. Framing breast cancer risk as an evolving process that reflects changes in underlying risk factors over time may help manage expectations and mitigate distress, particularly when risk estimates decrease and screening intensity is reduced [22, 23].

This study has several limitations. The study population was necessarily curated because women for whom IBIS risk recalculation was no longer applicable (e.g., those who developed breast cancer, underwent mastectomy, were older than 85 years in 2024, or were found to carry pathogenic mutations) were excluded from the baseline (2014) cohort. This may have introduced selection bias and led to an underrepresentation of high-risk women, as those who developed breast cancer during the 2014–2024 study period were excluded. Nevertheless, this cohort represents a subgroup of women with clinically elevated breast cancer risk for whom supplemental screening guidelines are not well established. The supplemental screening strategy evaluated in this study reflects current Belgian clinical practice and resource availability; therefore, the results may not be directly applicable to settings with different screening protocols or broader access to breast MRI.

Evaluating this population of women with dense breasts and a positive family history, but without a known pathogenic genetic mutation, provides valuable insights into risk stratification and screening approaches in a group for whom screening recommendations remain heterogeneous. This targeted approach acknowledges that women participating in breast cancer screening are not a homogeneous group and supports ongoing efforts toward risk-adapted rather than uniform screening strategies.

IBIS-based risk assessment relied on retrospective EPF data, which may contain inaccuracies or omissions due to self-reporting. In addition, risk calculations in 2014 and 2024 were performed by multiple researchers, which may have introduced inter-observer variability.

We compared the IBIS BC-LTR scores between 2014 and 2024 only, as the 10-year risk scores were not calculated or recorded in 2014. Furthermore, only IBIS v8.0b has been tested as an epidemiological breast cancer risk model, where the model also has inherent limitations [12].

Future studies should explore how artificial intelligence (AI)-based risk models and short-term risk estimation tools may complement traditional lifetime risk models like IBIS in the future, potentially further optimising personalised screening strategies, but are not yet routinely implemented in clinical practice [24–26].

In conclusion, IBIS BC-LTR estimates change over time in women with clinically elevated risk. Known risk factors such as high breast density, HRT use, increased BMI and family history of breast cancer were reconfirmed and proved to significantly influence IBIS BC-LTR changes over time. Periodic reassessment of IBIS BC-LTR may improve alignment between estimated risk and supplemental screening strategies; however, these findings should not be interpreted as prospectively predicting risk changes in broader unselected populations.

## Data Availability

The datasets generated and/or analysed during the current study are not publicly available due to GDPR but are available from the corresponding author on reasonable request.

## Abbreviations

BC-LTR: Breast cancer lifetime risk
BMI: Body mass index
EPF: Electronic patient files
HBOC: Hereditary breast and ovarian cancer
HRT: Hormone replacement therapy
